# Probiotics and Cat Health: A Review of Progress and Prospects

**DOI:** 10.3390/microorganisms12061080

**Published:** 2024-05-27

**Authors:** Musu Zha, Shimin Zhu, Yongfu Chen

**Affiliations:** Key Laboratory of Dairy Biotechnology and Engineering, Ministry of Education, Inner Mongolia Agricultural University, Hohhot 010018, China; zsmyxtk@126.com

**Keywords:** cats, health, probiotics

## Abstract

Cats are increasingly favored as companion animals; their health has drawn widespread attention. Given the continuous improvements in the required living standards of both humans and animals, inflammatory bowel disease, allergies, diarrhea, constipation, periodontal disease, obesity, diabetes, and other health issues have become recognized as valid pet problems. Antibiotics are commonly used to treat pet diseases, greatly improving animal health. However, antibiotic abuse is common, especially when seeking to treat bacterial infections. Probiotics are beneficial microorganisms that may be directly ingested in food or as feed additives; they improve the intestinal microflora balance, enhance immunity, and ensure healthy growth. However, cat data are usually inferred from reports on dogs or humans; cat research remains preliminary in nature. Therefore, we here describe the current understanding of how probiotics improve cat health, facilitating the further development and application of probiotics for cats.

## 1. Introduction

With pets viewed as important family members and the number of pets increasing annually, the pet industry has witnessed rapid developments. A recent study found that households globally own 471 million pet dogs and 373 million pet cats—one in two households in the United States and one in four households in China own a dog or a cat [1]. Moreover, the rate of increase in the number of cats has exceeded that of dogs in recent years. Cats are becoming increasingly favored as one of the most important companion animals of humans. Cat health status is thus receiving increasing attention.

Given the increases in the living standards of both humans and their pets, cat inflammatory bowel disease (IBD), allergies, diarrhea, constipation, periodontal disease, obesity, diabetes, and other health problems have become issues of concern to cat owners. Antibiotics are commonly used to treat pet diseases, and indeed significantly improve pet health. However, antibiotic abuse is not uncommon, especially when seeking to treat bacterial infections [2]. Such abuse can trigger bacterial antibiotic resistance, and excessive dosages can disrupt the intestinal floral balance, rendering pets prone to constipation, diarrhea, and other gastrointestinal diseases that seriously endanger their health [3]. Therefore, when seeking to prevent and treat cat diseases, agents other than antibiotics are required. Many studies have confirmed that probiotics improve cat gastrointestinal function, enhance immune system performance, prevent oral diseases, and reduce obesity. In developed countries, cat probiotic preparations are becoming the preferred treatments for cat health issues.

**Figure 1:**
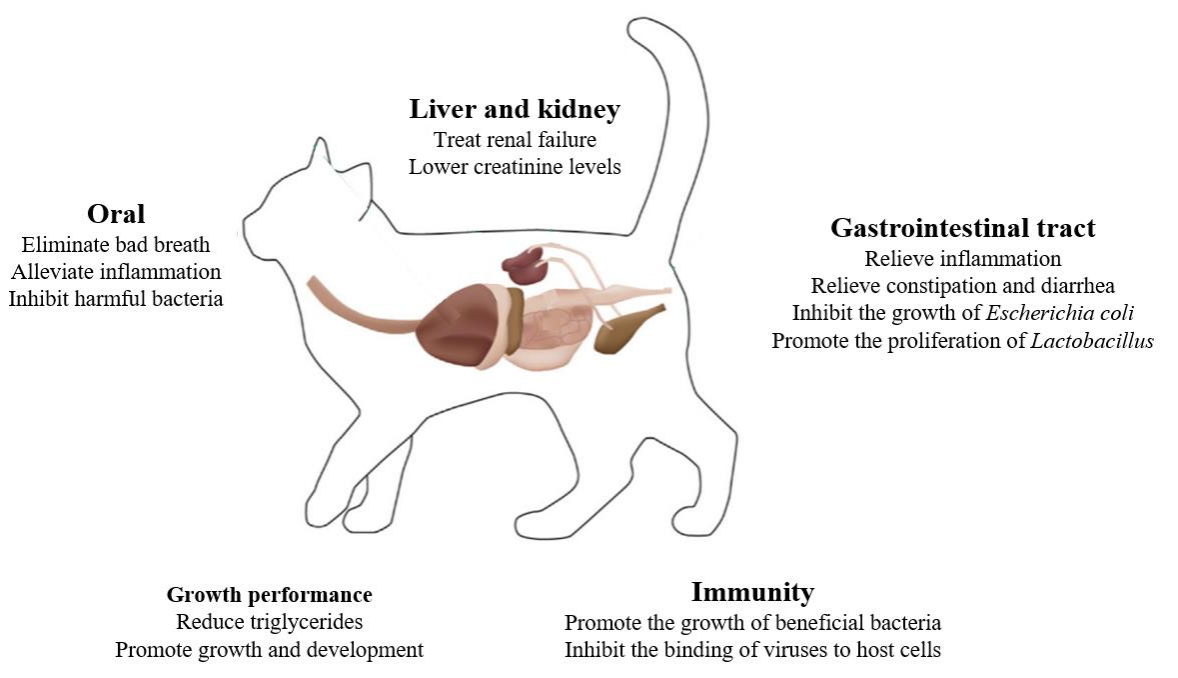
The roles of probiotics in cat health.

Probiotics are host-friendly active microorganisms, thus safe, nontoxic, devoid of residue accumulation, non-polluting, and incapable of developing drug resistance. Probiotics may be ingested in food or feed additives, and then improve the intestinal microecological balance, enhance immunity, and ensure healthy pet growth (Figure 1). Probiotics are becoming promising alternatives to antibiotics when seeking to optimize animal production. However, their effects on cats are often inferred from reports on dogs or humans. Cat research remains preliminary. Here, we review the current state of research on how probiotics affect cat health, facilitating the further development and application of probiotics for cats.

## 2. Probiotics and General Intestinal Health

The bacterial microorganisms of animal intestines are typically classified as symbiotic, pathogenic, or opportunistic; all microorganisms exhibit unique microbial balances [4]. The intestinal microbiota produce many metabolic products via fermentation; these include the short-chain fatty acids (SCFAs) that play crucial roles in the interactions between the host and pathogens [4]. The intestinal microbiota plays a vital role in animal health, affecting not only nutrient metabolism and absorption but also controlling host health. Gut microbiota dysbiosis may trigger both intestinal and systemic diseases, including IBD, allergies, constipation, obesity, diabetes, and kidney disease [5]. Chronic enteropathy (CE) is one of the most common gastrointestinal diseases of (particularly older) cats, and includes both IBD and small cell lymphoma. A significant decrease in the alpha diversity index and a decrease in the *Clostridium* abundance were observed in cats with CE [6]. Few diagnostic or treatment methods are yet available for cats with chronic gastrointestinal symptoms; most current treatment methods were derived for dogs [7]. Feline herpesvirus-1 (FHV-1) infection is common worldwide [8], very contagious, and often associated with severe clinical diseases in cats. The common clinical symptoms include fever, sneezing, nasal discharge, conjunctivitis, coughing, and difficulty breathing. Anti-FHV-1 vaccines do not prevent infection, and no drug yet eradicates FHV-1. Constipation is common in cats, defined by an absence, infrequency, or difficulty of defecation, associated with retention of fecal material in the colon and rectum [5,9]. Diarrhea is also common in cats, triggered by stress, dietary changes, and infections with various bacteria, viruses, and parasites [10]. Diarrhea is associated with excessive water in the feces. Pathophysiologically, diarrhea is divided into four types. However, in clinical practice, diarrhea is generally divided into parasitic, viral, bacterial, and other diarrhea based on the cause. Previous study compared the intestinal viral communities of clinically healthy cats and those with diarrhea and suggested that the *Astroviridae*, *Picornaviridae*, *Adenoviridae*, *Coronaviridae*, and *Picobirnaviridae* viruses may be the main causative pathogens of cat diarrhea [11]. Regardless of the cause, intestinal microbiota dysbiosis is apparent in both dogs and cats with diarrhea, and is closely related to diarrhea development.

In the clinic, antibiotics are commonly used to treat feline gastrointestinal diseases, significantly improving cat health. However, misuse has been associated with an increasing rate of antibiotic resistance accompanied by disorders of the cat gut microbiota and reductions in microbial diversity that severely compromise gastrointestinal health. Probiotics are commonly used to maintain gut health and have exhibited clinically valuable therapeutic effects in humans, thus somewhat reducing antibiotic misuse. However, the use of probiotics to treat cats remains exploratory. Probiotics effectively alleviate pet gastrointestinal diseases such as diarrhea, hepatic encephalopathy, ulcerative colitis, IBD, functional gastrointestinal disorders, and necrotizing enterocolitis [7]. Probiotics promote gut health by regulating the intestinal microbiome, secreting metabolites, including SCFAs and amino acids, and improving the antioxidant status. Given their beneficial effects on gut health, probiotics are increasingly incorporated into animal diets. Probiotic intervention studies in cats are summarized in Table 1. 

A previous study provided evidence supporting the improvement effect of *Saccharomyces boulardii* (10^10^ CFU/kg) and *Pediococcus acidilactici* (1.25 × 10^10^ CFU/kg) on cats’ health by modulating gut microbes and the production of metabolite SCFAs [4]. A closer study has revealed the addition of *Bacillus amyloliquefaciens* SC06 (10^10^ CFU/mL) and *Bacillus subtilis* B10 (10^10^ CFU/mL) to the diet of pet cats enhances the apparent digestibility of nutrients, and increases the total antioxidant capacity, glutathione peroxidase, and superoxide dismutase activity in serum, thereby improving health and reducing the incidence of diarrhea [12]. Lee et al. [13] found that *Bacillus licheniformis* fermentation products, previously used as additives in poultry and pig feed, improved fecal consistency in a few cats and significantly increased the numbers of *Blautia* spp., *Ruminococcus torques*, and *Ruminococcus gnavus* in feces. Wang et al. [14] discovered that probiotics (*Bacillus amyloliquefaciens* SC06 and *Bacillus subtilis* B10) significantly reduced the incidences of soft stools and diarrhea in pet cats, markedly increased the numbers of *Patescibacter* and *Plectosphaerella* in cat feces, and decreased the abundances of *Firmicutes*, *Gemmatimonadetes*, *Ruminococcaceae*, *Ascochytahe*, and *Saccharomyces*. The probiotic SLAB51™, comprising several *Lactobacilli*, *Bifidobacteria,* and *Streptococcus* species, significantly increased the numbers of *Lactobacillus* spp. (*p* = 0.03) and *Bacteroidetes* (*p* < 0.05) in cats, markedly improved the symptoms of constipation and idiopathic megacolon, and exhibited potential anti-inflammatory effects [9]. Bybee et al. [10] found that cats given the probiotic SF68 evidenced fewer diarrhea episodes within 2 days thereafter than did controls, indicating that probiotics may exert beneficial effects on the intestine. Similarly, Torres-Henderson et al. [15] found that the probiotic SF68 lowered the fecal scores and alleviated diarrhea. The *Escherichia coli* strain Nissle 1917 inhibited the in vitro growth of cat uropathogenic *E. coli* [16]. The probiotic strain *Enterococcus hirae* 1002-2 was found to decrease the intestinal permeability and fecal water loss caused by *E. coli* infection in certain specially bred kittens, and reduced diarrhea [17]. The addition of *Lactobacillus acidophilus* D2/CSL (CECT 4529) improved the fecal quality of healthy adult cats, thus enhancing gut health, increasing the number of *Lactobacillus* while reducing *Escherichia coli* counts [18]. Feeding of composite probiotics to pet cats significantly reduced the diarrhea rate, improved the digestibility of crude protein, and produced a 10.45% reduction in the number of *Escherichia coli* in the gut and an 8.77% increase of the *Lactobacillus* count [19]. *Bacillus subtilis* natto given daily to cats reduced gastrointestinal and bacterial symptoms including vomiting, diarrhea, and foul-smelling stool; improved gut function; allowed cats to recover from the decline in physical condition caused by the use of antibiotics and other drugs; enhanced immunity; and promoted growth [20]. *Lactobacillus reuteri* NBF 2 DSM 32264 improved the fecal quality parameters of healthy adult Persian cats, leading to an increase in *Lactobacillus* numbers (*p* < 0.05) and a decrease in the total coliform count (*p* = 0.011) [21]. *Lactobacillus plantarum* L11 may affect fat metabolism in cats by exerting a positive impact on the gut microbiome, thereby reducing odorous substances and improving nutrient digestion [22]. These preliminary findings indicate that probiotics may alter the gut microbiota by increasing beneficial bacteria and reducing harmful bacteria. Further research is needed to investigate the effects of these probiotic-based alterations to the gut microbiome on feline health, as well as other action pipelines.

## 3. Probiotics and Growth Performance

Kittenhood is a crucial stage in a cat’s life, characterized by rapid growth and vigorous metabolism. The cat growth curve reveals that most growth occurs between 3 and 6 months, and that 75% of the adult weight is attained by 7 months [23]. Adequate metabolism is essential for appropriate growth; diet, exercise, and emotional well-being all affect growth [24]. Kittens have an immature digestive system; nutrient absorption is weaker than that of adult cats and the nutritional needs of kittens and adults differ. Therefore, many adult cat food formulae are not suitable for kittens and may cause indigestion, and thus be poorly absorbed, if consumed. Addition of *Bacillus subtilis* natto to pet food effectively promoted cat growth and development [20]. The care method used affects the activity levels, habits, and emotional well-being of cats. Thus, it is important to choose appropriate care methods based on the specific needs of different cats [24].

Traditional cat litter clumps when it becomes moist. However, bacterial growth after clumping not only harms cat health but also can cause zoonotic diseases. Addition of antibacterial agents to cat litter significantly reduces clumping, associated with extensive waste when replacing the litter. Traditional cat litter contains deodorizing fragrances, but these fail to eliminate odors. Cats value warmth; self-heating cat litter is desirable. Probiotics exert beneficial effects on both humans and animals. Addition of complex probiotics (*Lactobacillus rhamnosus*, *Aspergillus niger*, *Bacillus subtilis*, and *Bacillus megaterium*) to cat litter competitively inhibits the growth of harmful bacteria, effectively preventing zoonotic diseases [25]. They have the added benefits of improving clumping, deodorization, and the warming performance of cat litter [25].

Obesity in cats is a serious issue; approximately 60% of cats are overweight or obese [26]. Various factors can contribute to obesity in cats, such as the gastrointestinal microbiome, genetics, neutering, reduced activity, and diets high in fat and energy, the most significant being excessive energy intake and storage [27]. Obesity hampers cat mobility and quality of life, reduces heat tolerance, predisposes cats to diseases, shortens the lifespan, reduces the efficiency of anesthesia, and triggers chronic inflammation [27,28,29,30,31,32,33]. Feline obesity is a complex but treatable condition. Weight can be managed by restricting energy intake and increasing energy expenditure [27,33]. The principal surgical treatments are sleeve gastrectomy and a jejunal-ileal bypass with omentopexy [32]. There are two types of medications to treat obesity: inhibitors of digestive system absorption such as anti-amylases, emetics, and appetite suppressants; and growth hormones and thyroxine, which increase metabolism. However, any medication has associated risks. Several studies have indicated differences in the gastrointestinal microbiome composition between obese and lean cats and dogs, and probiotics are reported as active microorganisms that colonize dogs and cats, helping to maintain the balance of the intestinal flora, regulating immunity, and promoting fat digestion and absorption [5,7]. *L. plantarum* L11 significantly reduces blood triglyceride levels (*p* < 0.05), and increases the abundance of *Bifidobacteria* (*p* < 0.05) [22]. However, as obesity has many causes and is often accompanied by complications, there is as yet no specific and effective probiotic treatment regime. Some studies have suggested that probiotics alone do not well-combat cat obesity; fiber should be added to the diet to increase satiety, and the protein and fat contents reduced [34]. Further research on probiotics in the context of feline obesity is required.

## 4. Probiotics and Oral Health

Dogs and cats do not brush their teeth and are very resistant to brushing by humans. Brushing is often associated with gum redness and swelling, and gingivitis. Reported to 68% of cats, dental disease is the most common disease in cats; the clinical symptoms include bad breath, loss of appetite, drooling, excessive scratching, oral bleeding, tooth loss, and facial asymmetry [5,35]. FHV, feline calicivirus (FCV), and feline chlamydia are common oral infectious agents in cats and are also the principal pathogens of feline infectious upper respiratory tract diseases [36]. The oral diseases of cats are divided into inflammatory and tumorous types. The former include periodontitis, tooth resorption, chronic gingivitis, and other conditions [35,37,38,39,40,41]. Periodontal disease is very common in domestic cats, characterized by the loss of periodontium such as gums, alveolar bone, periodontal ligaments, and dental cementum, culminating in tooth loss. Bacteria in the genera *Porphyromonas* and *Tannerella* are the principal periodontal disease pathogens of cats [42]. The condition associated with feline odontoclastic resorptive lesions (FORL) is a cat inflammatory oral disease of unknown etiology, and is commonly termed tooth resorption. Teeth are absorbed because of the destructive activities of odontoclast cells. Certain salivary cytokines serve as inflammatory biomarkers of the condition that leads to FORL [41,43]. Chronic cat gingivostomatitis is a severe immune system-mediated inflammatory disease of the oral mucosa, associated with microbial dysbiosis, but the pathogenesis remains unclear. FCV and FHV-1 virus infections may contribute to disease development, but feline immunodeficiency virus (FIV) does not [38,40]. Tumor-related oral diseases can be divided into two types: odontogenic tumors such as peripheral odontogenic fibromas, acanthomatous ameloblastomas, and peripheral giant cell granulomas; and non-odontogenic tumors such as squamous cell carcinomas [35,44].

Current treatments of oral cat lesions include professional tooth cleaning, tooth extraction, and extensive medical management with antibacterial, anti-inflammatory, immunosuppressive, and immunomodulatory agents [38]. Most management plans include antibiotic treatment, but bacterial culture or sensitivity testing are often not performed. Long-term use of antibiotics may promote bacterial resistance, rendering antibiotics ineffective. Vientos-Plotts et al. advocated the use of probiotics to treat diseases of the respiratory system [45]. When cats with stomatitis were given *Lactobacillus plantarum* for 2 weeks, the oral ulcers, pain, and inflammation were relieved, and the bad breath disappeared [46]. Mäkinen et al. [39] reported that a mixture of powdered *Streptococcus thermophilus* SP4, *Lactobacillus plantarum* 14D, and *Lactobacillus rhamnosus* SP1 inhibited the growth of bacteria that caused oral cat infections. Tang et al. [47] treated cats with chronic gingivostomatitis using a combination of traditional drugs and probiotics; the ulcerative surface in the oral cavity was reduced, and the rate of recurrent inflammation fell. *Lactobacillus acidophilus* LM0230 captured FCV in the bacterial peptidoglycan, effectively reducing viral titers [48]. Noda et al. [49] found that plant-derived *Lactobacillus plantarum* SN35 N secreted negatively charged exopolysaccharides (EPS) into the extracellular space; this inhibited the binding of viruses to host cells, effectively inactivating FCV, shortening the recovery time, and increasing the survival rate. Lappin et al. [8] also reported that fecal microbial diversity was maintained throughout the study in cats (infected with FHV-1) supplemented with *Enterococcus faecium* SF68.

## 5. Probiotics and Immunity

Cats are similar to humans in that the immune system is weaker in early life than later years of life, rendering them susceptible to various infectious diseases. Common infectious diseases of cats include cat flu, feline panleukopenia, and feline infectious peritonitis. These infectious diseases pose great risks to kittens; the morbidity and mortality rates are high. Currently, kittens are vaccinated against these diseases 2.5 to 3 months after birth. However, clinical cases of vaccine failure are common, partly because poor vaccine storage renders vaccines ineffective, and partly because cats produce insufficient antibodies after vaccination; they thus remain at risk of disease.

The intestine is the largest immune organ in the body and plays a crucial role in the immune defense against exogenous pathogens. If the intestine is damaged, health is severely affected. The intestinal mucosal immune system is the first line of defense against pathogenic microbial infections. Probiotics colonize the intestinal mucosa; regulate the intestinal microbiota both directly and via secreted metabolites; enhance mucosal barrier function; control cytokine production; increase the phagocytic capacity; improve intestinal function; promote the development and action of the immune system (thereby enhancing pet immunity); and inhibit the growth of pathogenic bacteria. *Lactobacillus* LM0230 reduced the FCV titer via peptidoglycan-mediated virus capture [48]. The *Bacillus subtilis* natto promoted the proliferation of beneficial intestinal bacteria, including *Lactobacillus*, *Enterococcus*, and *Bifidobacterium*, thereby enhancing immunity [20]. Noda et al. [49] found that plant-derived *Lactobacillus plantarum* SN35N secreted negatively charged EPS into the extracellular milieu, inhibiting the binding of viruses to host cells, effectively inactivating FCV, shortening the recovery time from disease, and increasing the survival rate.

## 6. Probiotics and Liver and Kidney Health

In recent years, the incidence of renal diseases in cats has increased, accompanied by a rise in mortality [50]. Common renal diseases include renal failure [51,52] and autosomal-dominant polycystic kidney disease [53]. Renal failure in cats often requires extensive treatment that further compromises cat health. The most serious conditions are acute kidney injury and chronic renal failure [52,54]. Renal failure treatment typically involves conservative medication; the surgical options include fluid therapy, peritoneal dialysis, hemodialysis, and kidney transplantation [54]. Conservative approaches feature gradual increases in drug doses. Typically, antihypertensives, antiproteinuric agents, phosphate-binders, antiemetics, and antacids are used to manage the clinical symptoms and long-term complications associated with renal failure. However, Western medicine treatments commonly used in clinical practice may cause certain side-effects. In practice, the availability of kidney transplantation and dialysis may be limited, and the benefits of such approaches remain unclear. Therefore, early prevention, detection, and treatment are the most effective ways by which to manage feline renal failure [52,54,55]. Palmquist et al. combined Kibow Biotics^®^ probiotics with medication when treating feline renal failure; the creatinine levels fell in 86% of affected cats, suggesting that probiotics may reduce the incidence of feline renal failure [56]. Sofyan et al. [57] found that administration of complex probiotics to male cats with cystitis effectively controlled the disease.

A change in liver size is often an indicator of liver disease [58]. Common cat liver diseases include liver fibrosis, poisoning, copper-related liver conditions, and fatty liver [59]. Feline hepatic lipidosis (FHL), also termed feline fatty liver syndrome, is the most common liver disease [60,61]. Triglycerides accumulate in liver cells because of abnormal metabolism of lipids, proteins, and other materials, increasing the liver weight and volume, followed by liver damage. Cats of all ages may develop FHL, which is caused by stress, pregnancy, drug side-effects, chronic poisoning, excessive nutrition, endocrine disorders, and other factors that unbalance hepatic lipid metabolism. Clinically, FHL manifests as anorexia, weight loss, jaundice, lethargy, vomiting, and pain over the liver. In rare cases, hepatic encephalopathy may develop. The large amounts of triglycerides in the liver cells of affected cats can damage the liver, impairing function and even causing death from liver failure [60,61,62,63,64]. *L. plantarum* L11 significantly reduced the blood triglyceride levels, increased the secretory immunoglobulin A (slg A) by 30.1%, and reduced the levels of indole and 3-methylindole, which could probable enhance the cat’s immunity and decrease liver burden [22].

Many scholars are exploring cat kidney and liver health, and various methods have been proposed for the treatment of diseases. However, probiotics have received little attention; further research is needed.

**Table 1:** Comprehensive summary of probiotics intervention studies in cats.

Probiotics (Trade Names, Manufacturers)	Doses	Animals (n)	Observed Outcomes	References
*Saccharomyces boulardii* *Pediococcus acidilactici*	2.25 × 10^10^ CFU/kg (1 × 10^10^ CFU/kg *S. boulardii*; 1.25 × 10^10^ CFU/kg *P. acidilactici*	12 healthy cats	Modulation of gut microbe levels; improvements in microbiota-derived SCFA production; reduction in inflammatory conditions; improved antioxidant status; facilitation of settlement of *Lactobacillus* and *Bacillus* species; reduced proportions of thick-walled bacteria/pseudomonads	Li et al., 2023 [4]
*Enterococcus faecium* SF68 NCIMB10415	5 × 10^8^ CFU/day	12 cats with chronic FHV-1 infections	Reductions in the prevalence of diseases associated with chronic FHV-1 infection	Lappin et al., 2009 [8]
SLAB51™ *Streptococcus thermophilus* DSM32245 *Lactobacillus acidophilus* DSM32241 *Lactobacillus plantarum* DSM32244 *Lactobacillus casei* DSM32243 *Lactobacillus helveticus* DSM32242 *Lactobacillus brevis* DSM27961 *Bifidobacterium lactis* DSM32246 *Bifidobacterium lactis* DSM32247	2 × 10^11^ lyophilized bacteria per 5 kg body weight	7 cats with chronic constipation; 3 cats with idiopathic megacolon; 10 healthy cats	Significant improvement in the clinical symptoms of constipation and idiopathic megacolon	Rossi et al., 2018 [9]
*Enterococcus faecium* strain SF68	2.1 × 10^9^ CFU/day	217 cats with diarrhea and 182 dogs with diarrhea	Reductions in diarrhea rates	Bybee et al., 2011 [10]
*Bacillus subtilis* SC06 *Bacillus coagulans* B10	3 × 10^9^ CFU/kg	20 healthy cats	Improvement of the apparent digestion rate; enhanced antioxidant capacity; promotion of weight gain; reduction in the incidence of diarrhea	Wang et al., 2022 [12]
*Bacillus licheniformis*-fermented products	1.1 mg/kg	8 cats with chronic diarrhea; 4 healthy cats	Relief of diarrhea	Lee et al., 2022 [13]
*Enterococcus faecium* strain SF68		34 cats given amoxicillin and clavulanate	Relief of diarrhea	Torres-Henderson et al., 2017 [15]
*Enterococcus hirae* (1002-2)	1 × 10^8^ CFU/day	130 weaned kittens	Reduction in the diarrhea rate	Gookin et al., 2022 [17]
*Lactobacillus acidophilus* CECT 4529	5 × 10^9^ CFU/kg	10 healthy cats	Improved fecal quality; increases in *Lactobacillus* numbers; reduction in *Escherichia coli* numbers	Fusi et al., 2019 [18]
*Lactobacillus reuteri* NBF 2 DSM 32264	5 × 10^9^ CFU/kg	12 healthy cats	Improved fecal quality parameters; increased *Lactobacillus* counts; fewer coliform bacteria	Belà et al., 2024 [21]
*Lactobacillus plantarum* L11	1 × 10^9^ CFU/kg	12 healthy cats	Reductions in blood triglyceride levels; fewer odorous substances in feces; increased nutrient digestion rate	Han et al., 2024 [22]
*Streptococcus thermophilus* SP4 *Lactobacillus plantarum* 14D *Lactobacillus rhamnosus* SP1	Uncounted	9 healthy cats 13 healthy dogs	Inhibition of pathogenic bacterial growth	Mäkinen et al., 2019 [39]
The VSL#3 probiotic	2.25 × 10^11^ CFU/day	6 healthy cats	Relief from respiratory diseases.	Vientós-Plotts et al., 2017 [45]
Probiotics contain: *Lactobacillus casei* *Lactobacillus rhamnosus* *Lactobacillus acidophilus* *Lactobacillus bulgaricus* *Bifidobacterium infantis* *Bifidobacterium breve* *Streptococcus thermophilus*	9.5 × 10^8^ CFU/day	1 cat with idiopathic cystitis	Relief of cystitis	Sofyan et al., 2020 [57]

## 7. Conclusions

As owners become increasingly concerned about pet health, maintenance of cat health has become increasingly important. Some scholars have described probiotics that enhance cat health. However, most research remains preliminary in nature. The health-promoting effects of probiotics have yet to be fully confirmed, and their mechanisms of action remain unclear. In terms of applications, although the prospects are wide-ranging, current cat probiotics are mainly feed additives or target functional health. Diagnostic and treatment applications are few in number. Commercialization of cat probiotic products requires further work. Future research should focus on how probiotics improve cat health, thus providing a theoretical basis for the development of microbial preparations that will aid the diagnosis and treatment of cat diseases

## Data Availability

Data is contained within the article.

## References

[1] Statista (2020). Number of Dogs and Cats Kept as Pets Worldwide in 2018: Ema Bedford. https://www.statista.com/statistics/1044386/dog-and-cat-pet-population-worldwide/.

[2] Candellone A., Badino P., Girolami F., Ala U., Mina F., Odore R. (2023). Dog Owners’ Attitude toward Veterinary Antibiotic Use and Antibiotic Resistance with a Focus on Canine Diarrhea Management. Animals.

[3] Zhang M., Mo R., Wang H., Liu T., Zhang G., Wu Y. (2023). Grape seed proanthocyanidin improves intestinal inflammation in canine through regulating gut microbiota and bile acid compositions. FASEB J..

[4] Li Y., Ali I., Lei Z., Li Y., Yang M., Yang C., Li L. (2023). Effect of a Multistrain Probiotic on Feline Gut Health through the Fecal Microbiota and Its Metabolite SCFAs. Metabolites.

[5] Wernimont S.M., Radosevich J., Jackson M.I., Ephraim E., Badri D.V., MacLeay J.M., Jewell D.E., Suchodolski J.S. (2020). The Effects of Nutrition on the Gastrointestinal Microbiome of Cats and Dogs: Impact on Health and Disease. Front. Microbiol..

[6] Marsilio S., Pilla R., Sarawichitr B., Chow B., Hill S.L., Ackermann M.R., Estep J.S., Lidbury J.A., Steiner J.M., Suchodolski J.S. (2019). Characterization of the fecal microbiome in cats with inflammatory bowel disease or alimentary small cell lymphoma. Sci. Rep..

[7] Salavati S. (2024). Prebiotics, Probiotics and Faecal Transplants in Cats: Where Are We Now?. https://www.research.ed.ac.uk/en/publications/prebiotics-probiotics-and-faecal-transplants-in-cats-where-are-we/.

[8] Lappin M.R., Veir J.K., Satyaraj E., Czarnecki-Maulden G. (2009). Pilot study to evaluate the effect of oral supplementation of *Enterococcus faecium* SF68 on cats with latent feline herpesvirus 1. J. Feline Med. Surg..

[9] Rossi G., Jergens A., Cerquetella M., Berardi S., Di Cicco E., Bassotti G., Pengo G., Suchodolski J.S. (2018). Effects of a probiotic (SLAB51™) on clinical and histologic variables and microbiota of cats with chronic constipation/megacolon: A pilot study. Benef. Microbes.

[10] Bybee S.N., Scorza A.V., Lappin M.R. (2011). Effect of the probiotic *Enterococcus faecium* SF68 on presence of diarrhea in cats and dogs housed in an animal shelter. Vet. Intern. Med..

[11] Yi S. (2019). Viral Metegenomic Analysis of Feline Fecal Virome and Molecular Detection and Evolution Analysis of Feline Diarrhea-associated Viruses in Northeastern China. Ph.D. Thesis.

[12] Wang F., Li X., Xv S., Jin X., Xu C., Mei X., Li W. (2022). Effects of compound *Bacillus* on growth, nutrient apparent digestibility and health of pet cats. Chin. J. Anim. Nutr..

[13] Lee T.-W., Chao T.-Y., Chang H.-W., Cheng Y.-H., Wu C.-H., Chang Y.-C. (2022). The effects of *Bacillus licheniformis*—Fermented products on the microbiota and clinical presentation of cats with chronic diarrhea. Animals.

[14] Wang F., Mei X., Wang Q., Zhao P., Zhou Y., Tang L., Wang B., Xu S., Li X., Jin Q. (2023). Compound *Bacillus* alleviates diarrhea by regulating gut microbes, metabolites, and inflammatory responses in pet cats. Anim. Microbiome.

[15] Torres-Henderson C., Summers S., Suchodolski J., Lappin M.R. (2017). Effect of *Enterococcus faecium* strain SF68 on gastrointestinal signs and fecal microbiome in cats administered amoxicillin-clavulanate. Top. Companion Anim. Med..

[16] Snell C.B., Winston J.A., Quimby J.M., Diaz-Campos D., Gibson J.F., Harrison A., Byron J.M., Justice S.S., Rudinsky A.J. (2022). *Escherichia coli* probiotic exhibits in vitro growth-limiting effects on clinical feline uropathogenic *E coli* isolates. Am. J. Vet. Res..

[17] Gookin J.L., Strong S.J., Bruno-Bárcena J.M., Stauffer S.H., Williams S., Wassack E., Azcarate-Peril M.A., Estrada M., Seguin A., Balzer J. (2022). Randomized placebo-controlled trial of feline-origin *Enterococcus hirae* probiotic effects on preventative health and fecal microbiota composition of fostered shelter kittens. Front. Vet. Sci..

[18] Fusi E., Rizzi R., Polli M., Cannas S., Giardini A., Bruni N., Marelli S.P. (2019). Effects of *Lactobacillus acidophilus* D2/CSL (CECT 4529) supplementation on healthy cat performance. Vet. Rec. Open.

[19] Liu J. (2023). Compound probiotics and their impact on the intestinal health of pet cats. China Anim. Ind..

[20] Shi C. (2024). Effects of *Bacillis subtilis* natto on dogs and cats. Guangdong Feed.

[21] Belà B., Di Simone D., Pignataro G., Fusaro I., Gramenzi A. (2024). Effects of *L. reuteri* NBF 2 DSM 32264 consumption on the body weight, body condition score, fecal parameters, and intestinal microbiota of healthy persian cats. Vet. Sci..

[22] Han B., Liang S., Sun J., Tao H., Wang Z., Liu B., Wang X., Liu J., Wang J. (2024). The effect of *Lactobacillus plantarum* on the fecal microbiota, short chain fatty acids, odorous substances, and blood biochemical indices of cats. Microorganisms.

[23] Merenda M.E.Z., Sato J., Scheibel S., Uemoto A.T., Rossoni D.F., Santos M.P.D., Pereira L.C., Ribeiro L.B., Vasconcellos R.S. (2021). growth curve and energy intake in male and female cats. Top. Companion Anim. Med..

[24] Gao A., Chen J., Wang D., Liang S., Yuan B. (2023). Effects of different feeding methods on growth performance and blood indexes of british-shorthair cats. Lab. Anim. Sci..

[25] Wang C., He R., Dong G. (2022). Comparative experiment on quality evaluation and deodorization antibacterial effect of probiotic bentonite cat litter. China Anim. Health.

[26] He S., Ding L., Xu J., Zhao H., Chen N., Liu Q., Han L., Zhang H., Si B. (2023). Advancements in research on the relationship between human health and feline gut microbiota. China Feed.

[27] Liu F. (2020). Exploring scientific feeding and management techniques for obese dogs and cats. China Anim. Health.

[28] Caro-Vadillo A., Montoya-Alonso J.A., García-Guasch L. (2022). Impact of obesity on lung function in cats with bronchoconstriction. Vet. Sci..

[29] Martins T.D.O., Ramos R.C., Possidonio G., Bosculo M.R.M., Oliveira P.L., Costa L.R., Zamboni V.A.G., Marques M.G., de Almeida B.F.M. (2022). Feline obesity causes hematological and biochemical changes and oxidative stress—A pilot study. Vet. Res. Commun..

[30] Ma X., Brinker E., Graff E.C., Cao W., Gross A.L., Johnson A.K., Zhang C., Martin D.R., Wang X. (2022). Whole-Genome Shotgun Metagenomic Sequencing Reveals Distinct Gut Microbiome Signatures of Obese Cats. Microbiol. Spectr..

[31] Guo L., Zhang Z., Luo Y., Zhang Y., Zhu R., Zhang D., Li Q. (2022). Advancements in the study of anesthesia for obese dogs and cats. Chin. J. Vet. Med..

[32] Liu K. (2018). Causes and treatment of obesity in pet cats. Chin. J. Tradit. Vet. Sci..

[33] Wang S., Zhang S., Zhang L., Li J., Li T., Cai W., Zhang B., Qi Z. (2023). Research progress on the relationship between intestinal microbiota and obesity in dogs and cats. Chin. J. Anim. Sci..

[34] Chen B., Jiang B., Duan W., Liu T., Xu J., Huan Z., Zhang H. (2024). Research progress of probiotics on nutrition and health of dogs and cats. Feed Ind..

[35] Falcão F., Faísca P., Viegas I., de Oliveira J.T., Requicha J.F. (2020). Feline oral cavity lesions diagnosed by histopathology: A 6-year retrospective study in Portugal. J. Feline Med. Surg..

[36] Chan I., Dowsey A., Lait P., Tasker S., Blackwell E., Helps C.R., Barker E.N. (2023). Prevalence and risk factors for common respiratory pathogens within a cohort of pet cats in the UK. J. Small Anim. Pract..

[37] Anderson J.G., Rojas C.A., Scarsella E., Entrolezo Z., Jospin G., Hoffman S.L., Force J., MacLellan R.H., Peak M., Shope B.H. (2023). The oral microbiome across oral sites in cats with chronic gingivostomatitis, periodontal disease, and tooth resorption compared with healthy cats. Animals.

[38] Krumbeck J.A., Reiter A.M., Pohl J.C., Tang S., Kim Y.J., Linde A., Prem A., Melgarejo T. (2021). Characterization of oral microbiota in cats: Novel insights on the potential role of fungi in feline chronic gingivostomatitis. Pathogens.

[39] Mäkinen V.-M., Mäyrä A., Munukka E. (2019). Improving the health of teeth in cats and dogs with live probiotic bacteria. J. Cosmet. Dermatol. Sci. Appl..

[40] Older C.E., Gomes M.D.O.S., Hoffmann A.R., Policano M.D., Reis C.A.C.D., Carregaro A.B., Ambrósio C.E., Carregaro V.M.L. (2020). Influence of the fiv status and chronic gingivitis on feline oral microbiota. Pathogens.

[41] Thomas S., Lappin D.F., Bennett D., Nile C., Riggio M.P. (2024). Elevated pro-inflammatory cytokines and chemokines in saliva of cats with feline odontoclastic resorptive lesion. Res. Vet. Sci..

[42] Zhang X. (2022). The Relationship between Cat Periodontal Diseaseand Oral Flora and Its Prevention and Treatment. Master’s Thesis.

[43] Thomas S., Lappin D.F., Nile C.J., Spears J., Bennett D., Brandt B.W., Riggio M.P. (2021). Microbiome analysis of feline odontoclastic resorptive lesion (FORL) and feline oral health. J. Med. Microbiol..

[44] Reddy S.V., Renzi A., De Bonis P., Morandi L., Lenzi J., Tinto D., Rigillo A., Bettini G., Bellei E., Sabattini S. (2019). Prevalence of p53 dysregulations in feline oral squamous cell carcinoma and non-neoplastic oral mucosa. PLoS ONE.

[45] Vientós-Plotts A.I., Ericsson A.C., Rindt H., Reinero C.R. (2017). Oral probiotics alter healthy feline respiratory microbiota. Front. Microbiol..

[46] Liang S., Zhong Y., Wang J., Han B. (2023). Biological functions of lactic acid bacteria and lts research progress in clinical application in canine and feline. Chin. J. Vet. Med..

[47] Yang G. (2023). Advancements in research regarding the application and role of probiotics in promoting pet health, preventingand treating diseases. J. Jilin Agric. Univ..

[48] Aboubakr H.A., El-Banna A.A., Youssef M.M., Al-Sohaimy S.A.A., Goyal S.M. (2014). Antiviral effects of *Lactococcus lactis* on feline calicivirus, a human norovirus surrogate. Food Environ. Virol..

[49] Silva L.A., Neto J.H.P.L., Cardarelli H.R. (2019). Exopolysaccharides produced by *Lactobacillus plantarum*: Technological properties, biological activity, and potential application in the food industry. Ann. Microbiol..

[50] Xie Z. (2023). Diagnosis and treatment measures for chronic kidney failure in dogs and cats. Today Anim. Husb. Vet. Med..

[51] He C., Pang H., Wang S., Lin J. (2022). A case report of feline chronic renal failure treated by traditional Chinese veterinary medicine. J. Tradit. Chin. Vet. Med..

[52] Wang C. (2022). Clinical Investigation and Diagnosis of Acute and Chronic Kidney Disease in 93 Cats. Master’s Thesis.

[53] Yu Y., Shumway K.L., Matheson J.S., Edwards M.E., Kline T.L., Lyons L.A. (2019). Kidney and cystic volume imaging for disease presentation and progression in the cat autosomal dominant polycystic kidney disease large animal model. BMC Nephrol..

[54] Zhou C. (2019). Therapeutic Effect of Complex Amino Acid and Probiotics on Feline Kidney Injury. Master’s Thesis.

[55] De Santis F., Boari A., Dondi F., Crisi P.E. (2022). Drug-dosing adjustment in dogs and cats with chronic kidney disease. Animals.

[56] Palmquist R.E. (2006). A preliminary clincial evaluation of kibow biotics, ® a probiotic agent, on feline azotemia. J. Am. Holistic Vet. Med. Assoc..

[57] Sofyan M., Rosman N., Krisnu B., Kamaludeen J., Dadi T.B., Pertiwi H. (2020). Management of feline idiopathic cystitis (fic) using probiotic combination treatment. Indian Vet. J..

[58] An G., Kwon D., Yoon H., Yu J., Bang S., Lee Y., Jeon S., Jung J., Chang J., Chang D. (2019). Evaluation of the radiographic liver length/11th thoracic vertebral length ratio as a method for quantifying liver size in cats. Vet. Radiol. Ultrasound.

[59] Tomaszewska E., Yamkate P., Gold R.M., Twedt D.C., Suchodolski J.S., Steiner J.M., Lidbury J.A. (2022). Assessment of the intracellular distribution of copper in liver specimens from cats. PLoS ONE.

[60] Tang X., Ceng J., Zhou M. (2022). A case report of feline hepatic lipidosis secondary to triaditis. Fujian J. Anim. Husb. Vet. Med..

[61] Zhu P., Liang W. (2022). Diagnosis and treatment of feline hepatic lipidosis: A case report. Yunnan J. Anim. Sci. Vet. Med..

[62] Li Y., Chen X., Wang J., Ma Y. (2023). Diagnosis and treatment of feline hepatic lipidosis: A case study. Gansu Anim. Husb. Vet. Med..

[63] Wang Z. (2020). Correlation between Serum Biochemical Indexes Andimaging Changes in Rats And Cats with Fatty Liver. Master’s Thesis.

[64] Ying J. (2018). Diagnosis and Treatment of Fatty Liver in Cats. Master’s Thesis.

